# *Aspergillus Oryzae* S2 α-Amylase Domain C Involvement in Activity and Specificity: *In Vivo* Proteolysis, Molecular and Docking Studies

**DOI:** 10.1371/journal.pone.0153868

**Published:** 2016-04-21

**Authors:** Mouna Sahnoun, Sonia Jemli, Sahar Trabelsi, Leila Ayadi, Samir Bejar

**Affiliations:** 1 Laboratory of Microbial Biotechnology and Engineering Enzymes (LMBEE), Centre of Biotechnology of Sfax (CBS), University of Sfax, Sidi Mansour Road Km 6, P.O. Box 1177, Sfax, 3018, Tunisia; 2 Preparatory Institute for Engineering Studies, Sfax (IPEIS), University of Sfax, MenzelChaker Road Km 0.5, P.O. Box 3018, Sfax, Tunisia; Centro de Biología Molecular Severo Ochoa (CSIC-UAM), SPAIN

## Abstract

We previously reported that *Aspergillus oryzae* strain S2 had produced two α-amylase isoforms named AmyA and AmyB. The apparent molecular masses revealed by SDS-PAGE were 50 and 42 kDa, respectively. Yet AmyB has a higher catalytic efficiency. Based on a monitoring study of the α-amylase production in both the presence and absence of different protease inhibitors, a chymotrypsin proteolysis process was detected *in vivo* generating AmyB. *A*. *oryzae* S2 α-amylase gene was amplified, cloned and sequenced. The sequence analysis revealed nine exons, eight introns and an encoding open reading frame of 1500 bp corresponding to AmyA isoform. The amino-acid sequence analysis revealed aY371 potential chymotrypsin cleaving site, likely to be the AmyB C-Terminal end and two other potential sites at Y359, and F379. A zymogram with a high acrylamide concentration was used. It highlighted two other closed apparent molecular mass α-amylases termed AmyB_1_ and AmyB_2_ reaching40 kDa and 43 kDa. These isoforms could be possibly generated fromY359, and F379secondary cut, respectively. The molecular modeling study showed that AmyB preserved the (β/α)_8_ barrel domain and the domain B but lacked the C-terminal domain C. The contact map analysis and the docking studies strongly suggested a higher activity and substrate binding affinity for AmyB than AmyA which was previously experimentally exhibited. This could be explained by the easy catalytic cleft accessibility.

## Introduction

Amylases cover about 25 to33% of the world enzyme market [1]. They are used in several industries mainly in the hydrolysis of starch to generate glucose, maltose, a mixture of malto-oligosaccharides and α-limit dextrin-containing α-(1–6) bonds [2]. Those products are highly important in a wide range of nutritional, cosmetic and pharmaceutical applications [3–5].α-amylases were classified into α-1, 4-glucan-4-glucanohydrolase, EC 3.2.1.1 [6] according to their mode of hydrolysis. On the other hand, the amino-acid residue sequence similarity analysis classifies α-amylases into glycoside hydrolase (GH) family13 which shares three domains (A, B, C) [7]. Domain A is the catalytic domain formed by a (β/α)_8_-barrel. It is often called the TIM-barrel and is generally formed by a set of sub-sites that bind the glucose monomers[8].Domain B is generally a long loop connecting the third β-strand to the third α helix forming a substrate binding cleft at the interface of domains A and B. Domain C is commonly formed by an antiparallel β-sheet connected to domain A. The emergence of new sequences that have lower homology and the same structure as family 13 (such as enzymes acting on the trehalose and sucrose) has led to the emergence of new families [9]. Hence, the concept of "Clan α-amylase" has appeared including families 13, 70 and 77 as well as more than 500 different sequences so far. These sequences share a catalytic domain, a barrel structure (β/α)_8_, a retention hydrolysis mechanism and three catalytic residues which are Asp (strand β4), Glu (strand β5) and Asp (strand β7) [10].

Amylyotic enzymes may coexist in multiple isoforms. Besides, their production is controlled by different extracellular parameters such as salt concentration [11], cultivation type [12],and protease action [13].In this context, Ravi-Kumar et al. [14,15]demonstrated that the autoproteolysis of the precursor α-amylase enzyme causes a secretion of three α-amylases by *Aspergillus niger*. The enzymatic properties of all α-amylase forms produced in this case have shown similar properties with differences in molecular weights. The amino-acid residue sequence analysis confirmed that the essential regions of catalysis and stability [13] were conserved. Amylases isoforms may also be the result of post-translational modification events or an expression of multigene families [16, 17].Accordingly, Boel et al.[18]studied two glucoamylases from *Aspergillus niger* namely G1 and G2 and came to the conclusion that although they are synthesized from two different mRNAs, these two glucoamylases are closely related. The number of those isoenzymes varies according to the amylase origin reaching two for barley [19–21] and *aziki bean* [22] and three for malted sorghumα-amylases[2]. Although some of them had a small molecular weight difference reaching ± 2 kDa among malted finger millet α-amylases [16], these isoforms were distinguished by a divergent physicochemical properties and amino-acid residue sequence identity.

Understanding the production mechanism of isoforms is very important for favoring the emergence of a particular form with distinct properties such as high specific activity, starch binding domain and thermostability.

We have recently reported that a small derivative α-amylase of the *A*. *oryzae* S2 called AmyB has an apparent molecular mass reaching 42 kDa. The derivative is a proteolytic hydrolysis resulting from carboxyl-terminal side of a complete form AmyA [23].

This study reported on the encoding *A*. *oryzae* S2 α-amylase gene molecular cloning. It investigated the α-amylase production in both the presence and absence of different protease inhibitors to probe the AmyA proteolysis catalysis site. The molecular modeling of isoforms was also examined. Accordingly, novel insights into the implication of the C-terminal domain in the specificity, stability of the *A*. *oryzae* S2 α-amylase were introduced for the first time.

## Materials and Methods

### Microorganisms, Media and Culture Growth Conditions

The *A*. *oryzae* S2 used in this study was previously isolated [23] and propagated into the PDA medium plates at 30°C.

M medium was used for *A*. *oryzae* S2 α-amylase production in the current work. The composition of this medium was as follows: M (g/L): Gruel 25, Urea 12.5, casein acid hydrolysate 12.5, peptone Hy-Soy 6.25, glycerol 6.25, KH_2_PO_4_ 5, (NH_4_)_2_SO_4_ 2.5, and MgSO_4_ 2.5 [24]. The initial pH was adjusted to 5.0.

For culture growth, a seven-day-old *A*. *oryzae* S2 mycelium was harvested from the plates, dislodged under aseptic conditions and then transferred to the M medium. The inoculum was maintained for 24 h at 25°C with a 250 rpm agitation speed. The α-amylase production was carried out by inoculating 100mL of the culture medium in a 500-mL flask adding 10% of the inoculum preparation. The culture conditions were fixed at 25°C and 250 rpm. The protease inhibitors containing Leupeptine, PhenylMethaneSulfonyl Fluoride (PMSF), pepstatine A,and TPCK (N α-p-Tosyl L- PhenylalanylChloromethyl Ketone) with a final concentration of 50μM, 500μM, 1 μM and 50 μM, respectively, were tested. The protease inhibitors were individually added each 6hof culture to study their effect on α-amylase isoforms production. All protease inhibitors, which were characterized by analytical grade and the highest purity available, were purchased from Sigma Chemical Co. (St. Louis, MO, USA).The α-amylase isoforms produced from crude extracts of flask incubation in the culture medium were detected by zymogram. Alpha-amylases found in the crude extract of the culture, which did not include inhibitors at the same time, were taken as control.

The *Escherichia coli* Top10 (Invitrogen, USA) was used for AmyA cloning. It was cultured at 37°C in Luria Bertani (LB) medium (1% [w/v] BactoTryptone, 0.5% [w/v] yeast extract, and 0.5% [w/v] NaCl).

### DNA and Sequence Manipulation

The molecular biology techniques were carried out as described in a previous study [25]. The genomic DNA extraction was performed as described by Sahnoun et al. [23].

The alignment of the NH2-terminal sequence of the purified AmyA and AmyB enzymes allowed the constitution of the AmyD primer. The reverse AmyR primer was established from the C-terminal degenerate homologous parts of *A*. *oryzae* α-amylases. The *A*.*oryzae* S2 α-amylase gene was amplified with PCR primers AmyD (5’-CCACAGAAGGCATTT**ATG**-3’) containing the start codon (in bold) and AmyR (5’-TGC**TCA**GGCGTAACAGAT -3’) containing the stop codon (in bold). The reaction mixture amplification (50 μL) consisted of Pfu DNA polymerase amplification buffer (1× final concentration), 10^−5^μM of both primers, 200 μM of dNTPs, 300 ng of DNA template (genomic DNA of *A*. *oryzae* S2 [23]) and 2 U of Pfu enzyme (Thermo Scientific, Foster City, CA, USA). The cycling parameters were 94°C for 4 min followed by 30 cycles at 94°C for 30 s, 52°C for 45 s and 72°C for 2 min with a final extension of 72°C for10 min. DNA sequencing was carried out by an automated DNA sequencer named ABI Prism 3100-Avant Genetic Analyser (Applied Biosystems, Foster City, CA, USA) with the Big-Dye terminator cycle sequencing kit recommended by the manufacturer (Amersham Pharmacia Biotech, Buckinghamshire, UK).The sequence analyses and comparisons were performed using the BioEdit program. Homology search was performed using the BLAST search algorithm.

### Homology Modeling and Docking Studies

The 3D structural models for AmyA and isoforms were generated using the Deep-View version 3.5.1 program [26] and the crystal structure of α-amylase from *Aspergillus niger*(PDB accession code 2GUY_A) [27]having a 99% sequence identity with AmyA. The appropriate A365 was mutated to V using the “mutation tool”. The elimination from the end to amino-acid residue positions at Y359, Y371 and F379for AmyB_1_, AmyB, and AmyB_2_, respectively, were done by the “remove selected residues tool”. The generated models were improved by energy minimization using 200 cycles of the steepest descents and 500 cycles of the conjugate gradient. The stereochemical quality of the selected model was evaluated using the PROCHECK program [28].ThePyMol molecular Graphics System (DeLano Scientific, San Carlos, 184 CA. http://www.pymol.org.) was used in order to visualize the constructed model structures and to generate graphical figures. The maltotriose which is a frequently-used ligand in the study of the ligand–α-amylase interaction [29] was docked into AmyA and AmyB. The contact maps of the AmyA and AmyB isoforms were visualized using CMView-1.1.1 program. A CASP7 prediction was used to compare the native structure AmyA with the truncated AmyB one. A Ca contact type, 8.0 Å distance cutoff and a Needleman-Wunsch pairwise sequence alignment were chosen. Docking was performed with Autodockvina [30]. The grid box of 70 x 20 x70 and 40 x 40 x40 points was used for AmyA and AmyB, respectively, with a spacing of 1.0 Å. The grid box center was put on x = 9.69, y = 16, and z = 4.56 and x = 16.9, y = 6.2 and z = 0.59, respectively, for AmyA and AmyB. Gasteiger charges were assigned to protein and ligand molecules. Exhaustiveness was set on 20. An additional blind docking was also performed with the use of the Swissdock web server (http://swissdock.vital-it.ch/) [31].

### Biochemical Analysis

#### Alpha-amylase and Chymotrypsin Standard Assays

α-Amylase was evaluated by the addition of 50 μL of appropriately diluted enzyme to 0.5 ml of 1% (w/v) starch solubilized in 0.1M acetate buffer (pH 5.6).The mixture was incubated for 30 min at 50°C. The released reduced sugar (glucose equivalent) was determined using the 3, 5-dinitrosalicylic acid method [32]. A separate blank was prepared for each sample to eliminate the non-enzymatic liberate of sugar.

For the measurement of α-chymotrypsin activity, ATEE (Nacetyl-L-tyrosine ethyl ester) was used as substrate. The changes in absorbance were followed at 237 nm in a reaction mixture of 3 mL containing 40 mMTris/HCl (pH 8.0), 50 mM CaCl_2_ and 0.5 mM ATEE [33].A unit of enzyme activity was defined as the amount of enzyme releasing 1 μmoL product per minute under the assay conditions.

#### Protein Quantification and Zymogram

Protein concentration was determined referring to Bradford method with bovine serum albumin as a standard [34]. The zymogram for α-amylase activity (12% and 20% concentrations) was performed using the same conditions of SDS-PAGE [35] except that the polyacrylamide gel did not contain SDS. Amylolytic activity was evaluated by placing the native gel into an agarose gel containing (1%) soluble starch earlier to 30 min incubation at 50°C. The agarose gel was then stained with iodine reagent (2%iodine in 0.2% potassium iodine) and α-amylase activities were detected as transparent bands on a dark blue background [14].

## Results and Discussion

In a previous research work [23], we biochemically studied two α-amylases produced by *A*. *oryzae* S2 designated by AmyA and a proteolytic degradation AmyB derivative which had an apparent molecular weight of 50 and 42kDa, respectively. The effect of temperature on the purified isoforms stability confirmed that they retained more than 50% of their activity after an incubation of 60 min at a temperature range of 40–50°C(Fig A, B in S1 File) [23].

In this study, we cloned the gene encoding *A*. *oryzae* S2 α-amylase in *E*. *coli* and sequenced it. We also studied the proteolysis process of α-amylase isoforms production and the molecular modeling to generate a possible structural explanation of AmyB properties.

### AmyA Proteolysis Site Probe

**Fig 1:**
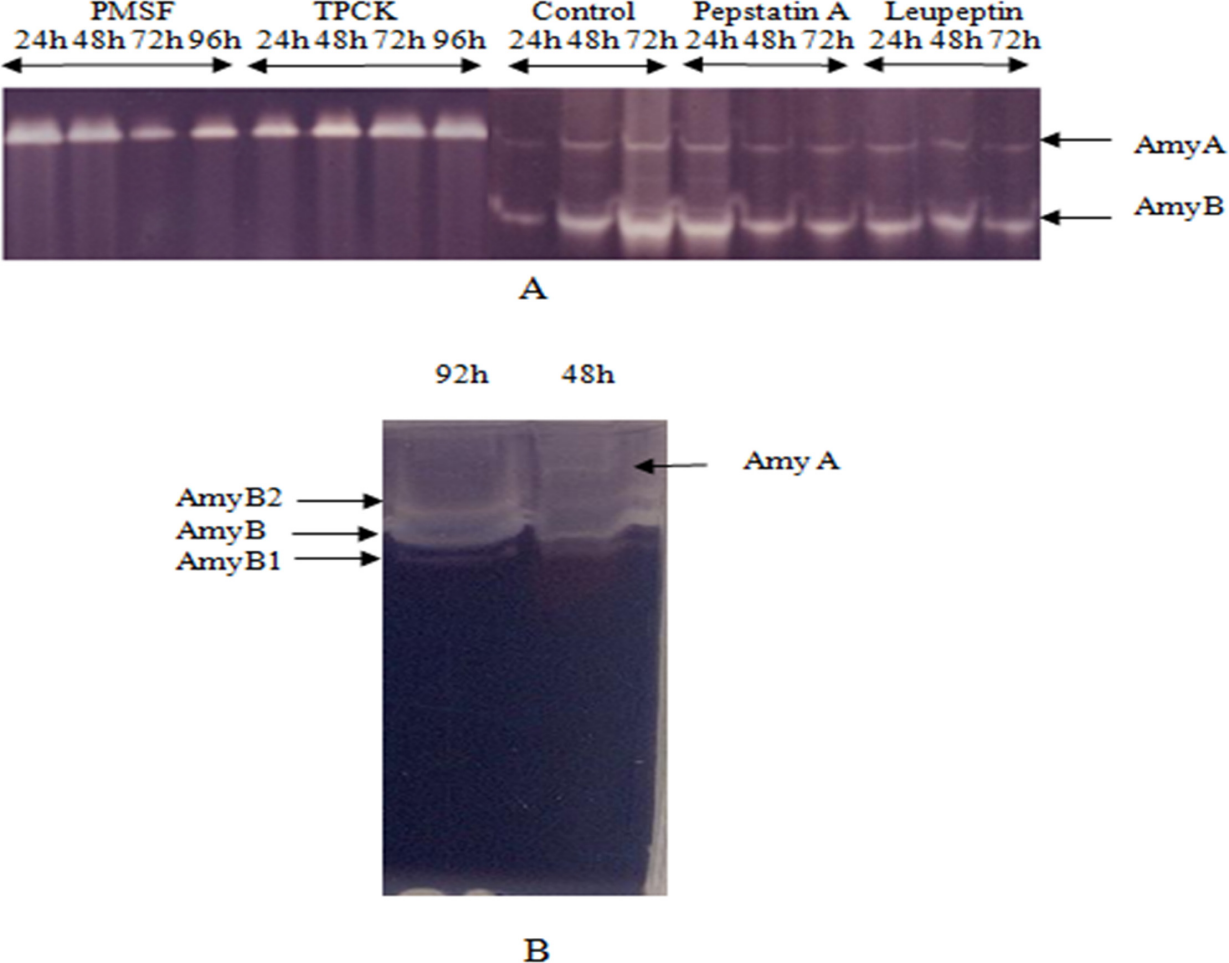
The zymogram of α-amylase activities. (A) The zymogram (10%) of α-amylase activities produced in the culture medium from the crude extract of flask incubation containing individually protease inhibitors Leupeptine, PMSF, pepstatine A as well as TPCK (N α-p-Tosyl L- PhenylalanylChloromethyl Ketone). The α-amylases produced in the culture medium from crude extracts without antiproteases were taken as control. (B) The zymogram (20%) of α-amylase activities of 48h and 92h of incubation time.

Seeing that AmyB is a current AmyA proteolytic product [23], a precise monitoring of α-amylase production in the presence and absence of different protease inhibitors was performed to elucidate the proteolysis process. Hence, several protease inhibitors including Leupeptine, PMSF, pepstatine A, and TPCK were individually added each 6h to the culture medium. The α-amylase isoforms produced during flask cultivation in the presence of each antiprotease were detected by zymogram (Fig 1A).

Alpha-amylase isoforms detected in the crude extract of the culture, which did not contain inhibitors at the sametime, were taken as control. In the presence of pepstatinA both isoforms were observed through the culture. Therefore, we concluded that the enzyme which was responsible for cleavage was not an aspartic proteaselike enzymes. However, in the presence of PMSF, only AmyA persisted throughout the time culture, suggesting that AmyB formation could be dependent on a serine or a cysteine protease action. When a Leupeptin was added, both isoforms were observed through the culture. Thus, the proteolytic hydrolysis was able to implement an α-chymotrypsin protease. To confirm this hypothesis, a chymotrypsin-like serine antiprotease, namely TPCK, was also individually tested as a protease inhibitor (Fig 1A). As a result, AmyA was not cleaved in the presence of TPCK and was noted to persist throughout the cultivation time. It was, therefore, possible to conclude that the proteolysis effect exerted on AmyA was due to *A*. *oryzae* S2 chymotrypsin protease. In order to confirm the presence of *A*. *oryzae* S2 chymotrypsin, the hydrolysis activity towards ATEE was investigated in the crude extract at different pH and temperature. The results confirmed the presence of the activity reaching 65 U/mL at an optimum pH equal to 8 and a temperature equal to 37°C. The existence of the proteolytic activity was already described for the other *A*. *oryzae* [36–38], for *A*. *fumigatus* [39] and for the other *A*. species [40–42].

### Molecular Cloning, Nucleotide, and Amino-Acid Sequence Analysis

The amplification of AmyA led to a unique 2.2 kb fragment (S2 File). The sequence analysis of the α-amylase genes showed that it was arranged as nine exons and eight introns. It shared an extensive homology (equal to a 99% of identity) with the genes encoding α-amylase from *Aspergillus oryzae*, *Aspergillus awamori*, *Aspergillus flavus*, *Aspergillus kawachii* and *Aspergillus niger* (Table 1).

**Table 1:** The sequence comparison of *Aspergillus oryzae* S2 AmyA with the existing α-amylase sequences provided by the Blast.

Microorganisms	DNA sequence identity (%)	Protein sequence identity (%)	Accession code	PDB code
*Aspergillus niger*	99	99	2GUY	2GUY
*Aspergillus oryzae*	99	98	AAA32708.1	2TAA
*Aspergillus kawachii*	99	99	dbj|AB109452.1|	**-**
*Aspergillus awamori*	99	99	BAD06002.1	-
*Aspergillus flavus*NRRL3357	99	99	XP002374124.1	-

**Fig 2:**
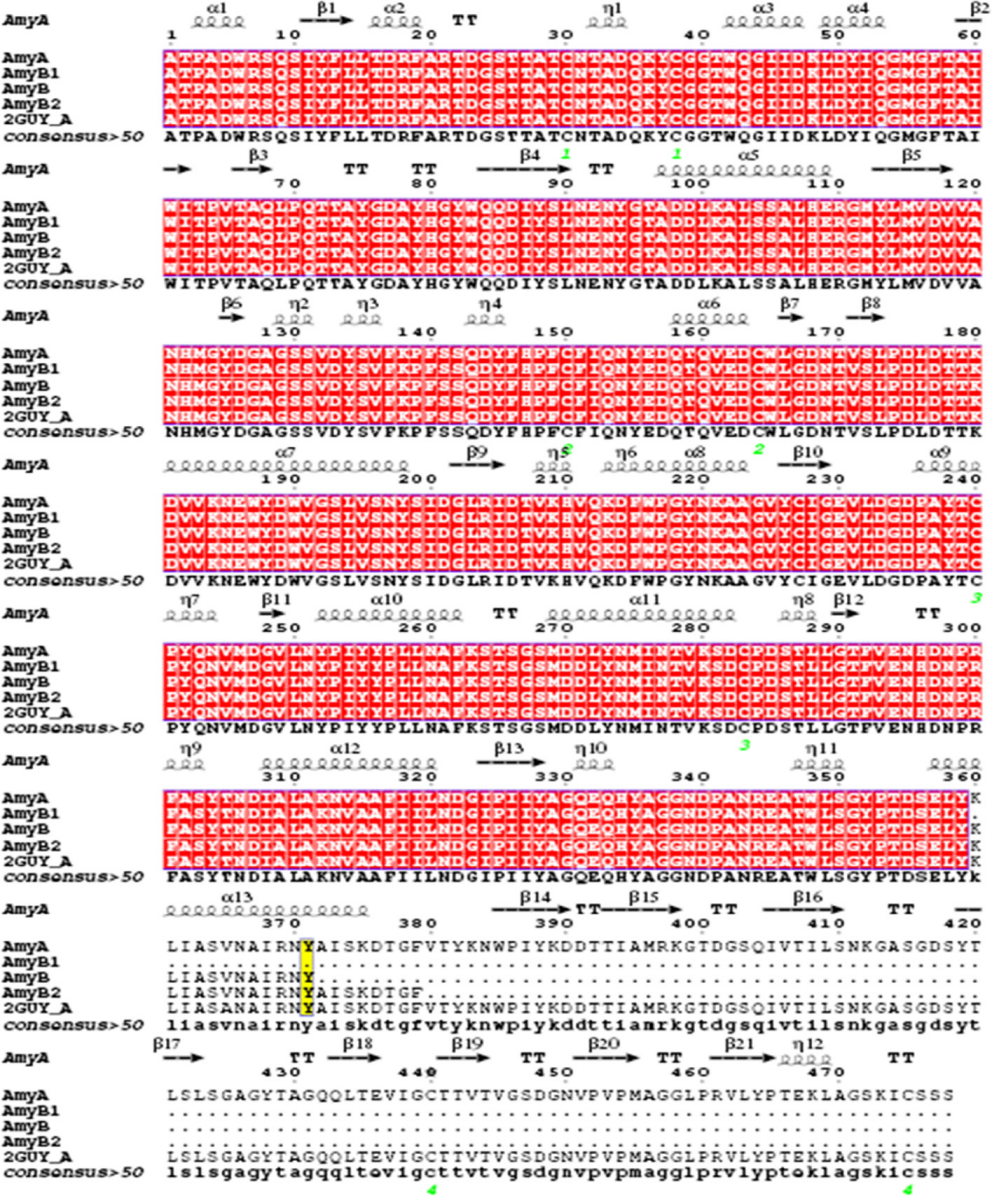
The Amino-Acid Sequence Analysis of *Aspergillus Oryzae* S2 α-amylases. (A, B) The Structure-based multiple sequence alignment of AmyA, AmyB_1_, AmyB, AmyB_2_, (Accession code Hx2000049571) of *Aspergillus oryzae* S2 with α-amylase of *Aspergillus niger* (Accession code 2GUY_A). The invariable residues among sequences are typed in white on a red background; differences between conserved groups are displayed on a yellow background; the numbers (1, 2, 3, and 4) correspond to the disulfide bonds.

The sequence analysis also revealed the presence of an upstream signal sequence consisting of 63 bp encoding 21 amino-acid residues (S2 File). The exons would encode an open reading frame of 1500 bp and 500 amino-acid residues of52.51 kDa calculated molecular mass (MM) which corresponded to the apparent AmyA molecular mass. The analysis of amino-acid sequence of AmyA (Fig 2) allowed the NH2-terminal sequence already determined to be identified[23].

The triad of catalytic residues corresponding to D206, D297 and E230 involved in catalysis was also identified. Based on the deduced amino-acid sequence as well as the corresponding MM of AmyA and AmyB and knowing that these two isoforms had the same NH2-terminal sequence, we could strongly predict the cleavage site. Hence, three possible catalysis sites at the AmyA C-terminal extremity, namely at Y359, Y371 and F379 amino-acid residue positions, could lead to the AmyB formation. These catalysis sites produced three isoforms of approximately calculated masses reaching 40.1 kDa, 41.5 kDa and 42.3 kDa. In agreement with this hypothesis, a zymogram of crude *Aspergillus oryzae* S2 extract was conducted under a concentrated acrylamide gel of20% to detect the possible closed MM isoforms presence. The result confirmed the presence of three isoforms namedAmyB_1_, AmyB, andAmyB_2_which had apparent molecular masses of 40 kDa, 42 kDa, and 43 kDa (Fig 1B). Four disulfide bonds were present in AmyA while only three S-S bonds were found in AmyB_1_, AmyB, and AmyB_2_. The fourth disulfide bridge missing in AmyB isoforms after AmyA proteolysis could explain the enhancement of the specific activity and the catalytic power (V_max_, K_cat_) of the truncated AmyB α-amylase (Table 2). In this context, the role of an extra disulfide bond on the activity of the cold-active α-amylase was investigated using a double mutant (Q58C/A99C) constructed on the basis of the 3D structure. The double mutant exhibited a two-fold lower specific activity with a clear trend to decrease kcat [43].

**Table 2:** The recapitulation of kinetic constants and general physico-chemical parameters of the AmyA and AmyB of *Aspergillus oryzae* S2 isoforms. Kinetic constants were previously evaluated [23]. Physico-chemical parameters revelation was performed using the Swiss-ProtParam tool (http://www.expasy.org/tools/).The instability index computed classified AmyA and AmyB as stable proteins.

**Kinetic constants**	**AmyA**	**AmyB**
K_m_ (mg mL^-1^)	4.70	2.60
V_max_ (Umg^-1^)	5303	8234
k_cat_ (s^-1^)	4419.10	5821.90
k_cat_ K_m_^-1^ (ml mg^-1^ s^-1^)	940.20	2205.20
Specific activity (U mg^−1^)	5620	6670
**Physico-chemical parameters**	**AmyA**	**AmyB**
Number of amino-acid residues	478	371
Theoretical pI	4.48	4.33
Total number of negatively charged residues (Asp + Glu)	54	46
Total number of positively charged residues (Arg + Lys)	30	21
Instability index	22.4	22.24
Formula	C_2344_H_3525_N_597_O_742_S_18_	C_1862_H_2750_N_470_O_583_S_14_
Extinction coefficients	106160	93085

### Molecular Modeling Studies

**Fig 3:**
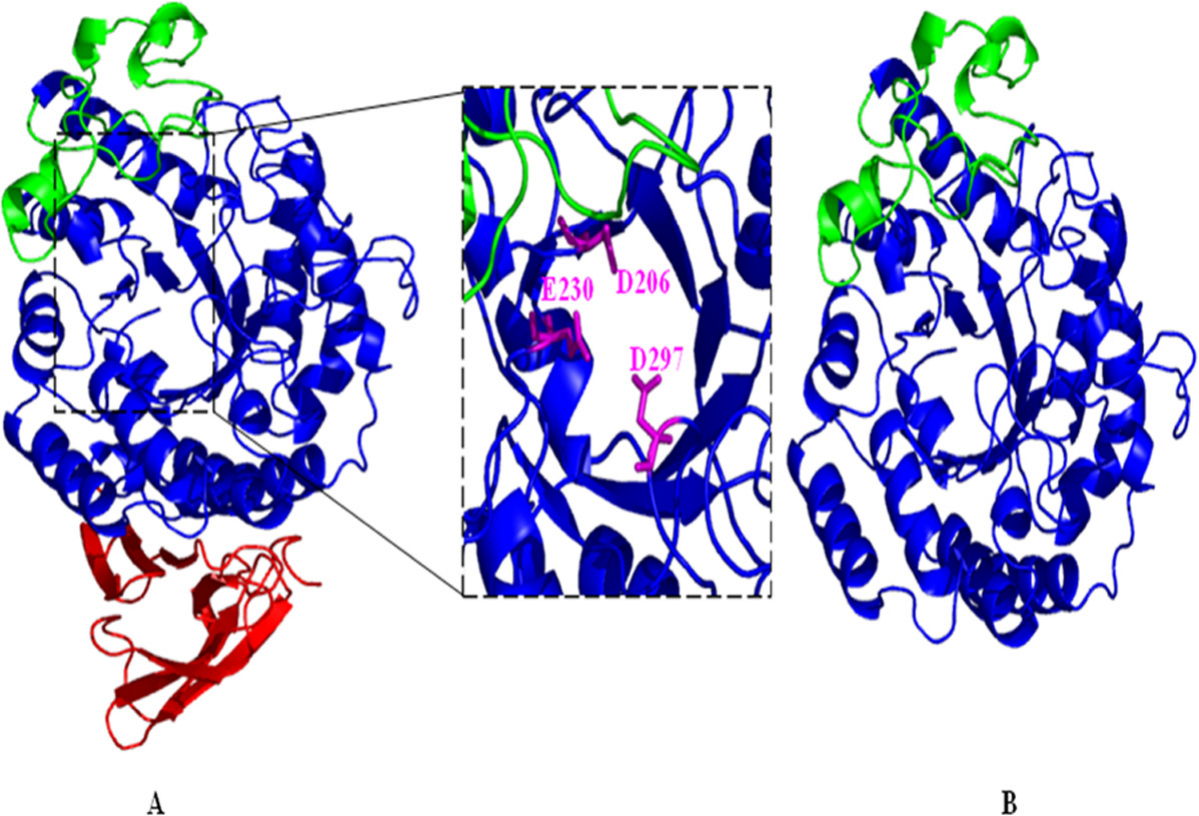
(A) Overview of the 3D model of AmyA (A) and the truncated AmyB (B) with SWISS-MODEL using *A*. *niger* α-amylase (PDB code: 2GUY) as a template. The individual domains are colored as follows: catalytic (β/α) _8_-barrel domain A blue, domain B green, domain C red. Close-up view of AmyA active site is represented. The triad residues are shown as maganta sticks. Graphical presentations were prepared using the PyMol software.

The sequence alignment of mature AmyA (478 residues) using psi-Blast showed high identity with *A*. *niger* α-amylase (PDB code: 2GUY_A) and *A*. *oryzae* Taka-α-amylase A (PDB code: 2TAA) reaching 99% and 98% with 100% of coverage. Compared to *A*. *niger* α-amylase, AmyA showed only a single substitution of V365A among 478 amino-acid residues. As commonly seen with fungal α-amylases, the structural model of the generated AmyA showed typically three domains (A, B and C) (Fig 3A)[7].

Domain A showed a central catalytic domain formed by a (β/α)_8_ TIM-barrel. Domain B was a small domain which lied between the strand β3 and helix α3 of the TIM-barrel while domain C was located at the C-terminal end and structured as eight antiparallel β-sandwich fold. A single conserved calcium binding site was identified in the structure of AmyA and found to bridge the A and B domains as in the case of all α-amylase structures. The presence of the calcium may have been important for stabilizing the loop conformation which formed a large lip over the substrate binding groove [44]. The three conserved catalytic amino-acid residues of the α-amylase family were also identified in the 3Dmodel (Fig 3) namely D206 which acted as the nucleophile, E230 which was the catalytic acid/base, and D297which was involved in the transition state. The analysis of the structural model of AmyB_1_, AmyB and AmyB_2_proved that the two essential domains of the enzyme catalysis including domain A and domain B were well conserved which explained the conservation activity in the truncated isoforms. Indeed, domain A and B may have played a major role in enzyme catalytic and in the stability of substrate binding process, respectively [8,45].The eliminated107residues in AmyB included the totality of domain C (Fig 3).The comparison of the physico-chemical parameters of AmyA and AmyB isoforms using the Swiss-ProtParam tool (http://www.expasy.org/tools/) including theoretical pI and instability index showed a close similitude (Table 2).

**Fig 4:**
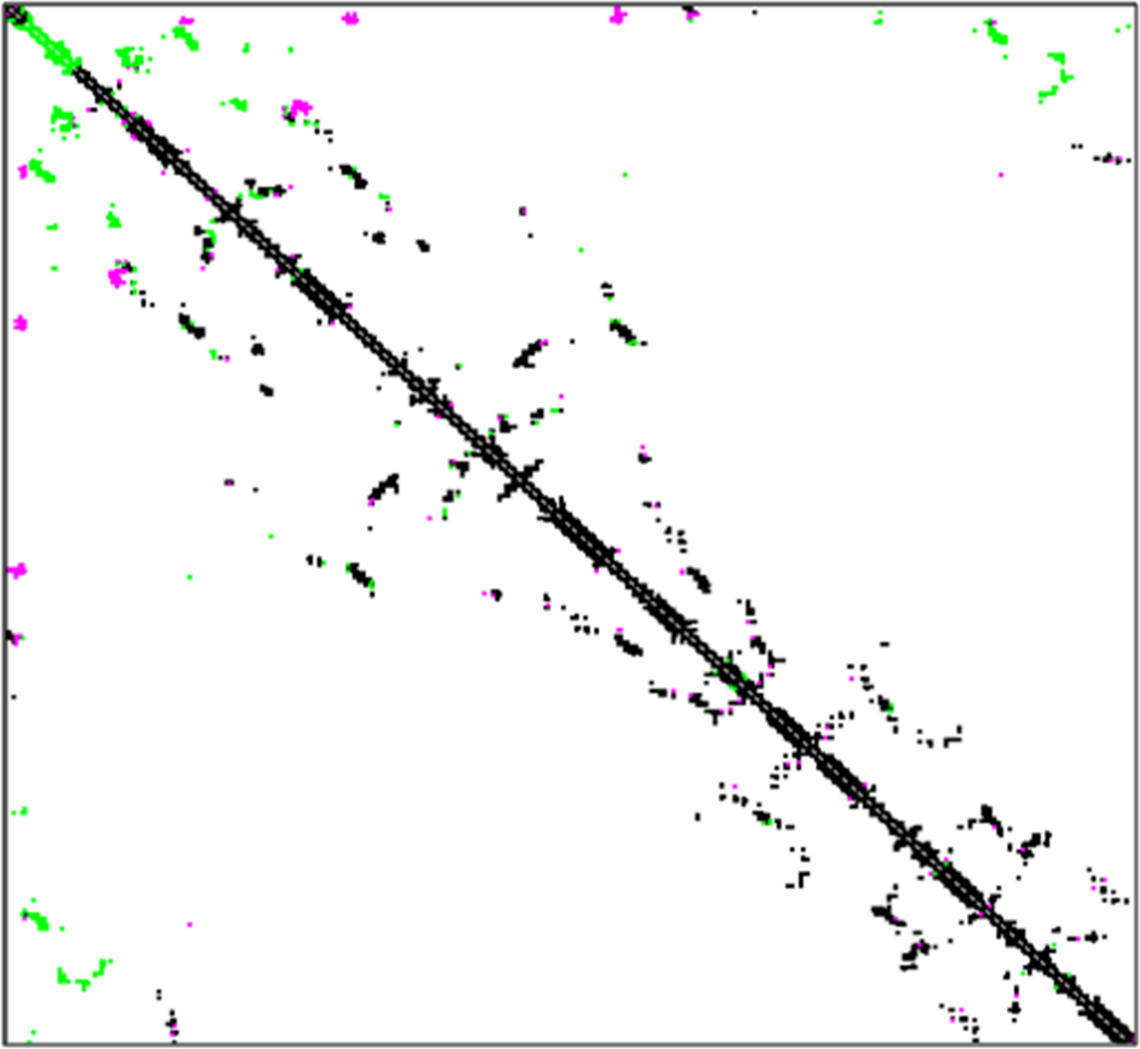
The contact maps of AmyA and AmyB. The black dots show the common contacts, the pink dots show the contacts which are unique to the native structure and the green for the contacts unique to the truncated enzyme structure.

The instability index classified these enzymes as stable proteins. To investigate the proteolysis effect on the tertiary structure of an enzyme, a contact map was used for the visualization and comparison of network of contacts among amino-acid residues belonging to the native and truncated AmyB protein (Fig 4).

A contact map was a particularly useful 2D representation of a protein 3D structure that could reflect the stability of the tertiary structure of the enzyme. The black dots showed the common contacts, the pink dots showed the contacts unique to the native structure and the green dots showed the contacts unique to the truncated enzyme structure. As shown in Fig 4, there was an obvious distinction in contacts in the catalytic domain. The existence of more green dots in the catalytic domain demonstrated a more compact structure of AmyB than AmyA. A more compact structure in this domain was a criterion of higher activities [46].On the other hand, compacted structures were necessary for the catalytic activity of α-amylase [47]. Therefore, it seemed that the activity of the native form was reduced in comparison with the truncated enzyme. This result was in agreement with the experimental observation [23]. Generally, the overview of other truncated α-amylase contact maps (result not shown) revealed that the pink and green dots were more marked for AmyB_1_, AmyB and AmyB_2_. The highest amounts of the pink and green dots showed the advanced difference in the compared structures. These results indicated that the changes in the tertiary structure of the truncated AmyB_2_were the lowest, and therefore the most stable conformation structure among the other truncated enzymes.

### Docking Studies

**Fig 5:**
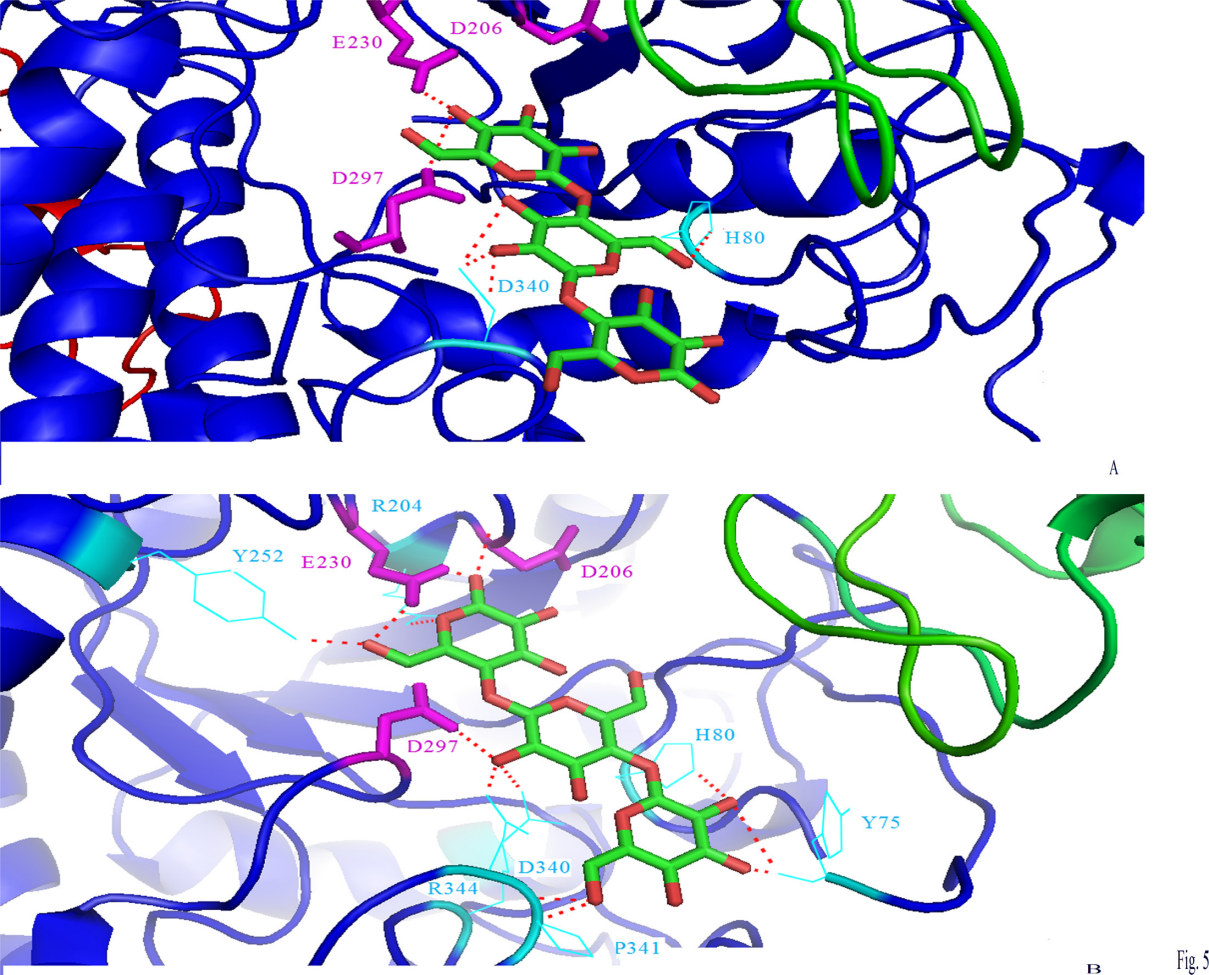
The binding sites of maltotriose on the AmyA (A) and AmyB(B) enzymes. The interactions between maltotriose and the amino-acid residues are showed by the dashed red lines. The catalytic residues were represented by a labeled maganta sticks, the other aminoacids implicated in the interaction are represented by a labeled cyan line. Domains A, B, and C are represented with blue, green, and red, respectively.

The enzymes were docked with maltotriose. The binding sites of maltotriose on the native enzyme and the truncated one are shown in Fig 5. D206, E230, and D297 (shown in sticks) in the binding site interacted with ligand. These residues coincided with the proposed catalytic triad of α-amylase in experimental and simulation studies [29, 47–48]. It was clearly seen that the binding site of maltotriose concerning AmyB exposed more polar contact number with ligand than AmyA (Fig 5).

Indeed, in addition to the catalytic residues interaction with the substrate, 7 other aminoacids comprising Y75, H80, R204, Y252, D340, P341, R344 were also involved in the AmyB-maltotriose complex. However, AmyA-maltotriose complex exposed just H80 and D340 (Fig 5). The atomic contact energy and the approximate interface area of the complex maltotriose-α-amylase for AmyA and AmyB were 19.44, 37.31 kcal/mol and 492.4 and 616.6 Å^2^, respectively. Therefore, seeing that AmyB exhibited more binding affinity than AmyA, it was more active.This result agreed well with our experimental studies showing that the affinity of AmyB to soluble starch (K_m_ = 2.60 mg/mL) was about 2 folds higher than the native type (K_m_ = 4.7 mg/mL). This observable fact could also be explained by the easy accessibility of the substrate to the catalytic cleft in the absence of domain C which lacked its common form in GH 70[49]. This domain was also called DUF1966 with an unknown function according to the Pfam database [50]. The beginning of the DUF1966 domain corresponds to the PIYKDD conserved sequence which was at 386 position of amino-acid sequence (Fig 2).The increase of AmyB activity contrasted with the trend of the studied C-terminal truncated α-amylases. Indeed, many experimental observations showed that the truncation of α-amylase from C-terminal did not change the amylolytic activity of the enzyme [51–52, 53]. In addition, the deletion of 90 amino acids of *Klebsiella pneumoniae* CGTase from C-terminal led to a truncated one with a similar activity of the native form [54]. Furthermore, other researchers reported that the first 410 amino acids of *Lactobacillus amylovorus* were sufficient for catalytic activity and specificity [55]. The elimination of 186 C-terminus amino acids of the *B*. *subtilis* X23 α-amylase did not affect the catalytic activity, the specificity pattern, the transglycosylation ability, the pH-activity and stability, the optimum temperature, and the binding ability to the starch [56]. However, the 10–13 amino acids elimination from C-terminal of Alkaliphilic *Bacillus* CGTase generated an enzyme with more activity in starch than that of the native enzyme. It also led to a reduced thermal stability [57]. Furthermore, Mehta et al. [58] and Iefuji et al. [59] reported that the domain C in *Bacillus* played a role in the thermostability of α-amylases [59].

We previously demonstrated that in addition to AmyA and AmyB already monitored in submerged culture, this strain produced an AmyB oligomeric form, in particular, a dominant tetrameric form named AmyC [12].The electrostatic potential maps and energies were evaluated for AmyA and AmyB (S3 File). AmyB possessed a slightly more electrostatic potential energy. A more in-depth analysis will be the objective of our future study to find a precise explicative proof for the intermolecular interaction and oligomerization process of AmyB.

## Conclusions

This study was undertaken to describe, for the first time, a chymotrypsin proteolysis generating three α-amylase isoforms AmyB_1_, AmyB and AmyB_2_ of 40 kDa, 42 kDa, 43 kDa that were revealed by a zymogram with a high acrylamide concentration. We demonstrated that AmyA isoform was encoded by an open reading frame of 1500 bp that was deduced from the cloned gene containing nine exons and eight introns. The deduced amino-acid sequence of the ORF revealed three potential chymotrypsin cleaving sites at Y371, Y359, and F379 likely to be AmyB_1_, AmyB, and AmyB_2_ C-Terminal end respectively. The molecular modeling study showed that AmyB conserved the (β/α)_8_ barrel domain and domain B but lacked the C-terminal domain C. The contact map analysis and the docking studies confirmed the high catalytic efficiency of AmyB as it was exhibited experimentally. This observable fact could be explained by the easy substrate accessibility to the catalytic cleft in the absence of domain C.

## Ethical Statements

“This article does not contain any studies with human participants or animals performed by any of the authors.”

## Supporting Information

S1 File The effects of temperature and pH on the stability of the purified AmyA and AmyB. The effect of temperature on the stability of the purified AmyA (A) and AmyB (B) at 40°C (■), 50°C (▲), 60°C (♦), and 70°C (X). (C) The effect of pH on the stability of the purified AmyA (▲) and AmyB (■). The residual activities were determined after the incubation of the enzymes at various pH values with the buffer solutions at 0.1 M (ranging between pH 3–12) and at 4°C for 48 h.
(TIF)

S2 File The DNA sequence of *Aspergillus oryzae* S2 AmyA. Exons and introns are shown with capital letters and a low case letter, respectively. The signal sequence is shown in blue and both the start and the stop codons are shown in red.
(TIF)

S3 File **The electrostatic potential maps of AmyA (A) and AmyB (B)** (red, negative; blue, positive; and white, neutral/hydrophobic potential).
(TIF)
