# Supplementary figures and images for: Aspergillus Oryzae S2 α-Amylase Domain C Involvement in Activity and Specificity: In Vivo Proteolysis, Molecular and Docking Studies
Source: PLoS One. 2016 Apr 21;11(4):e0153868. doi: 10.1371/journal.pone.0153868 (PMC4839703; doi:10.1371/journal.pone.0153868)

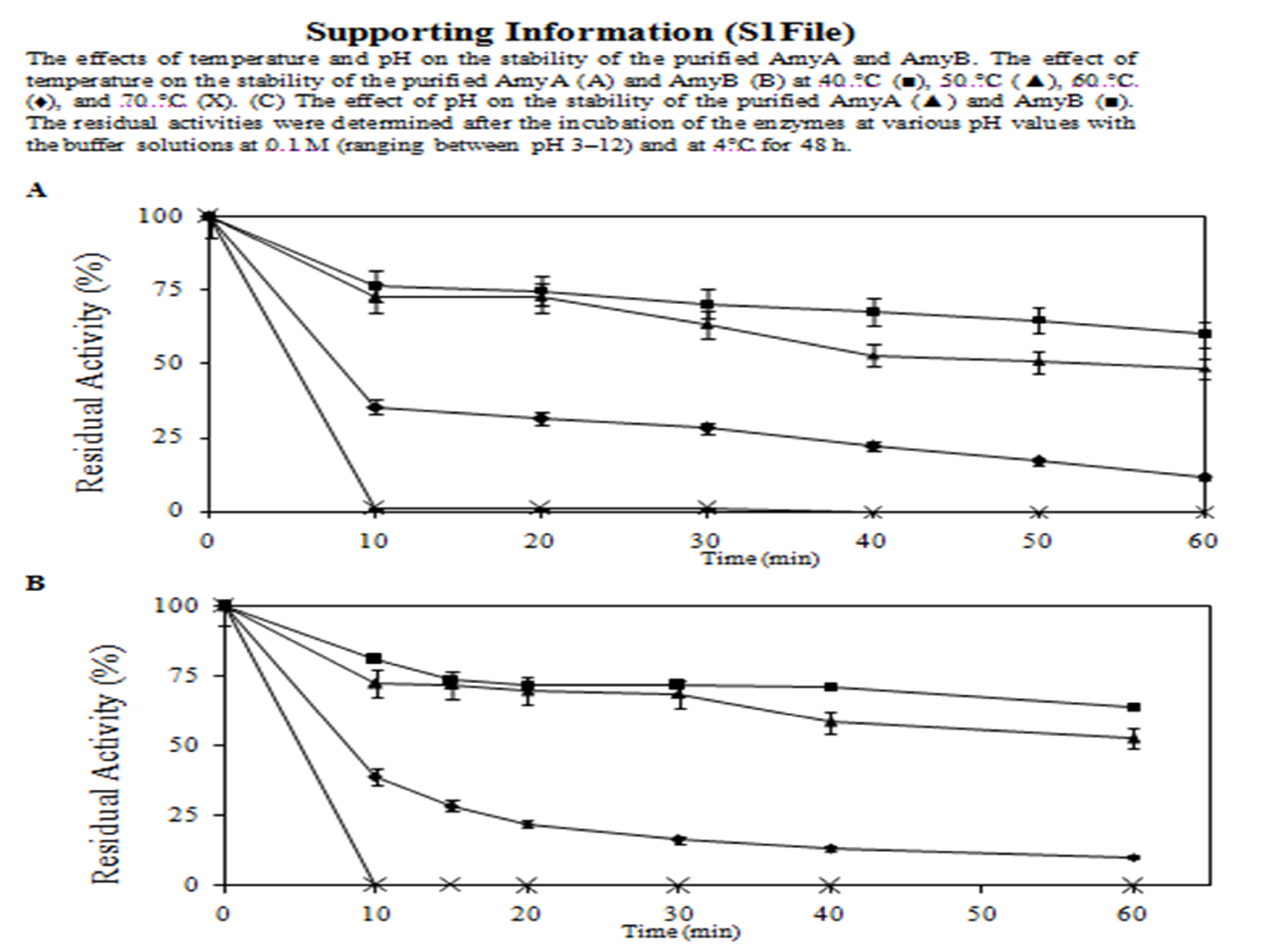

Supplement: S1 File — The effect of temperature on the stability of the purified AmyA (A) and AmyB (B) at 40°C (■), 50°C (▲), 60°C (♦), and 70°C (X). (C) The effect of pH on the stability of the purified AmyA (▲) and AmyB (■). The residual activities were determined after the incubation of the enzymes at various pH values with the buffer solutions at 0.1 M (ranging between pH 3–12) and at 4°C for 48 h. (TIF) [file pone.0153868.s001.tif]

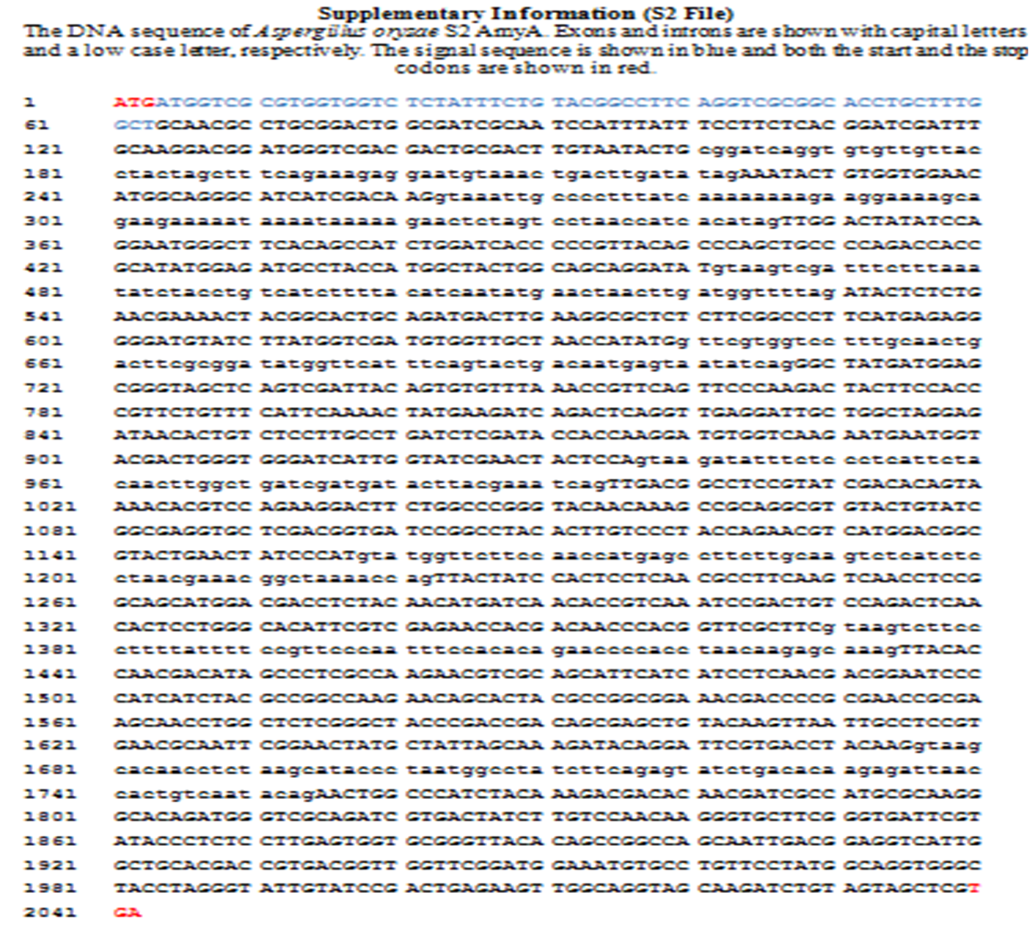

Supplement: S2 File — Exons and introns are shown with capital letters and a low case letter, respectively. The signal sequence is shown in blue and both the start and the stop codons are shown in red. (TIF) [file pone.0153868.s002.tif]

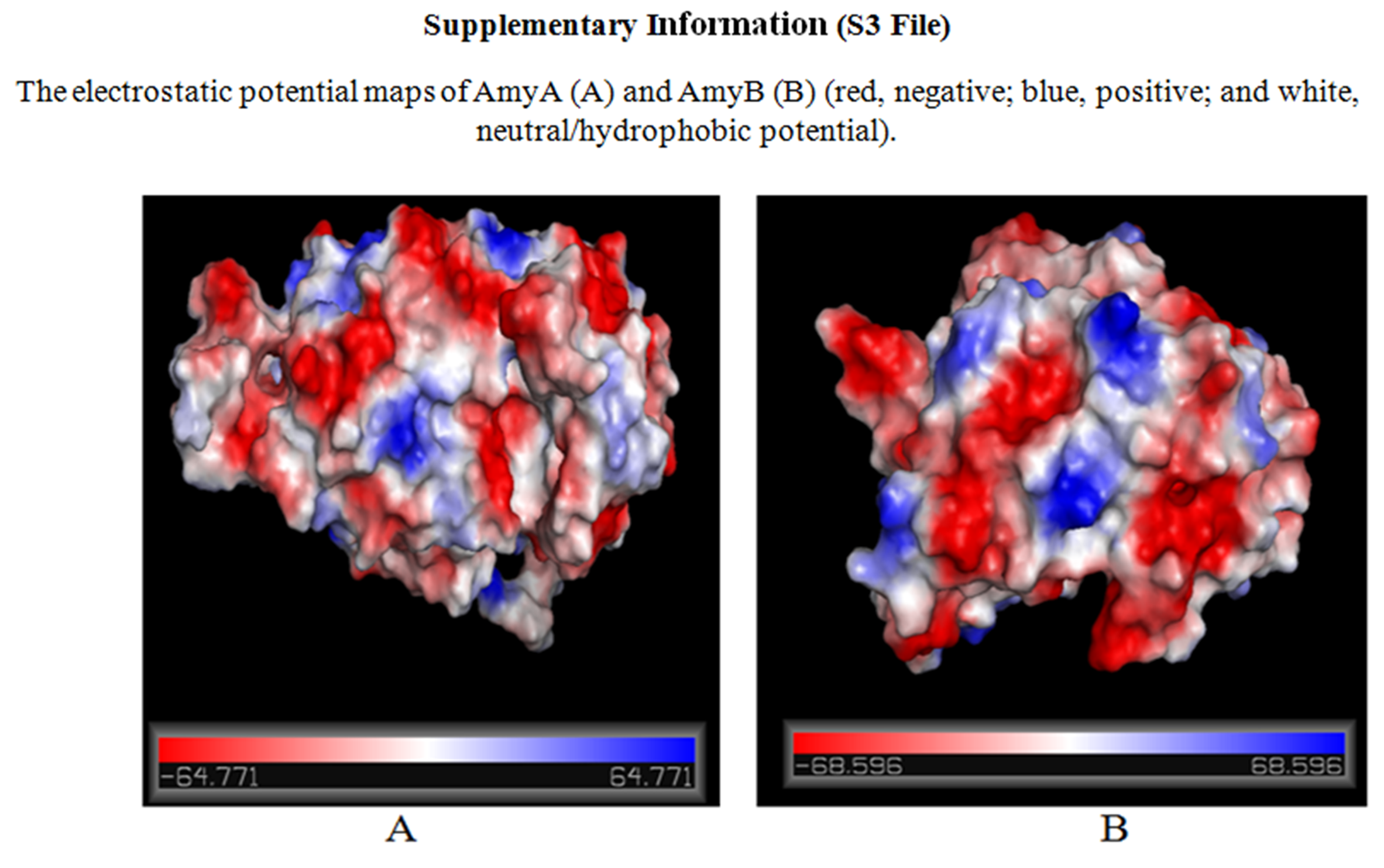

Supplement: S3 File — The electrostatic potential maps of AmyA (A) and AmyB (B) (red, negative; blue, positive; and white, neutral/hydrophobic potential). (TIF) [file pone.0153868.s003.tif]
